# Three-Dimensional Muscle Architecture and Comprehensive Dynamic Properties of Rabbit Gastrocnemius, Plantaris and Soleus: Input for Simulation Studies

**DOI:** 10.1371/journal.pone.0130985

**Published:** 2015-06-26

**Authors:** Tobias Siebert, Kay Leichsenring, Christian Rode, Carolin Wick, Norman Stutzig, Harald Schubert, Reinhard Blickhan, Markus Böl

**Affiliations:** 1 Department of Sport and Motion Science, University of Stuttgart, Stuttgart, Germany; 2 Institute of Motion Science, Friedrich-Schiller-University Jena, Jena, Germany; 3 Institut für Versuchstierkunde und Tierschutz, Universitätsklinikum Jena, Jena, Germany; 4 Institute of Solid Mechanics, Technical University at Braunschweig, Braunschweig, Germany; Ohio State University Medical Center, UNITED STATES

## Abstract

The vastly increasing number of neuro-muscular simulation studies (with increasing numbers of muscles used per simulation) is in sharp contrast to a narrow database of necessary muscle parameters. Simulation results depend heavily on rough parameter estimates often obtained by scaling of one muscle parameter set. However, *in vivo* muscles differ in their individual properties and architecture. Here we provide a comprehensive dataset of dynamic (*n* = 6 per muscle) and geometric (three-dimensional architecture, *n* = 3 per muscle) muscle properties of the rabbit calf muscles gastrocnemius, plantaris, and soleus. For completeness we provide the dynamic muscle properties for further important shank muscles (flexor digitorum longus, extensor digitorum longus, and tibialis anterior; *n* = 1 per muscle). Maximum shortening velocity (normalized to optimal fiber length) of the gastrocnemius is about twice that of soleus, while plantaris showed an intermediate value. The force-velocity relation is similar for gastrocnemius and plantaris but is much more bent for the soleus. Although the muscles vary greatly in their three-dimensional architecture their mean pennation angle and normalized force-length relationships are almost similar. Forces of the muscles were enhanced in the isometric phase following stretching and were depressed following shortening compared to the corresponding isometric forces. While the enhancement was independent of the ramp velocity, the depression was inversely related to the ramp velocity. The lowest effect strength for soleus supports the idea that these effects adapt to muscle function. The careful acquisition of typical dynamical parameters (e.g. force-length and force-velocity relations, force elongation relations of passive components), enhancement and depression effects, and 3D muscle architecture of calf muscles provides valuable comprehensive datasets for e.g. simulations with neuro-muscular models, development of more realistic muscle models, or simulation of muscle packages.

## Introduction

About 600 skeletal muscles enable complex movements like locomotion or laughing in humans. Musculoskeletal models with an increasing number of muscles have been used to investigate human and animal movements [1–4]. Although many phenomenological Hill-type [5–8] and biophysical [9, 10] muscle models have been developed to represent the dynamics of isolated muscles, the phenomenological modeling approach dominates in musculoskeletal modeling for simplicity and low computational cost. Because individual muscle parameters are lacking, a common approach in such studies is to scale one set of muscle parameters, e.g. by maximum isometric force and fiber length, to fit all muscles [2–4]. However, muscles differ in their individual muscle properties not only by scale, and these differences impact simulation results [11].

The active contractile properties of muscle fibers can be characterized by the hyperbolic force–velocity relation [5] and the force-length relation [12]. It has generally been found that slow twitch fibers exhibit a more bent force–velocity relation and lower maximum shortening velocity than fast ones [13]. Moreover, it is known that muscles exhibit a complex architecture consisting of fascicles with different lengths and pennation angles [14–16]. The specific architecture influences the shape of the muscle’s force-length relation [17]. Thus, simple scaling of these relations introduces errors of unknown magnitude. A broader database of lumped muscle model parameters for specific muscles would improve the situation; in parallel, development of more realistic three-dimensional (3D) muscle models requires individual muscle architecture as input [18–20].

Passive muscle properties vary considerably between muscles too [21]. For instance, passive forces of frog gastrocnemius arise already on the ascending limb of the force–length relation and reach about 30% maximum isometric force (*F*_*im*_) at optimal muscle length [22]. In contrast, passive forces of the frog semitendinosus muscle arise on the descending limb of the force-length relation [23]. Depending on muscle function tendons exhibit different mechanical properties [24], e.g. the tendon of the human wrist mover *M*. *extensor carpi radialis longus* used for positioning tasks is very stiff (1.8% strain at *F*_*im*_, [25]) compared with the more compliant human gastrocnemius tendon exploited for elastic recoil (5% strain at *F*_*im*_, [26]).

**Fig 1:**
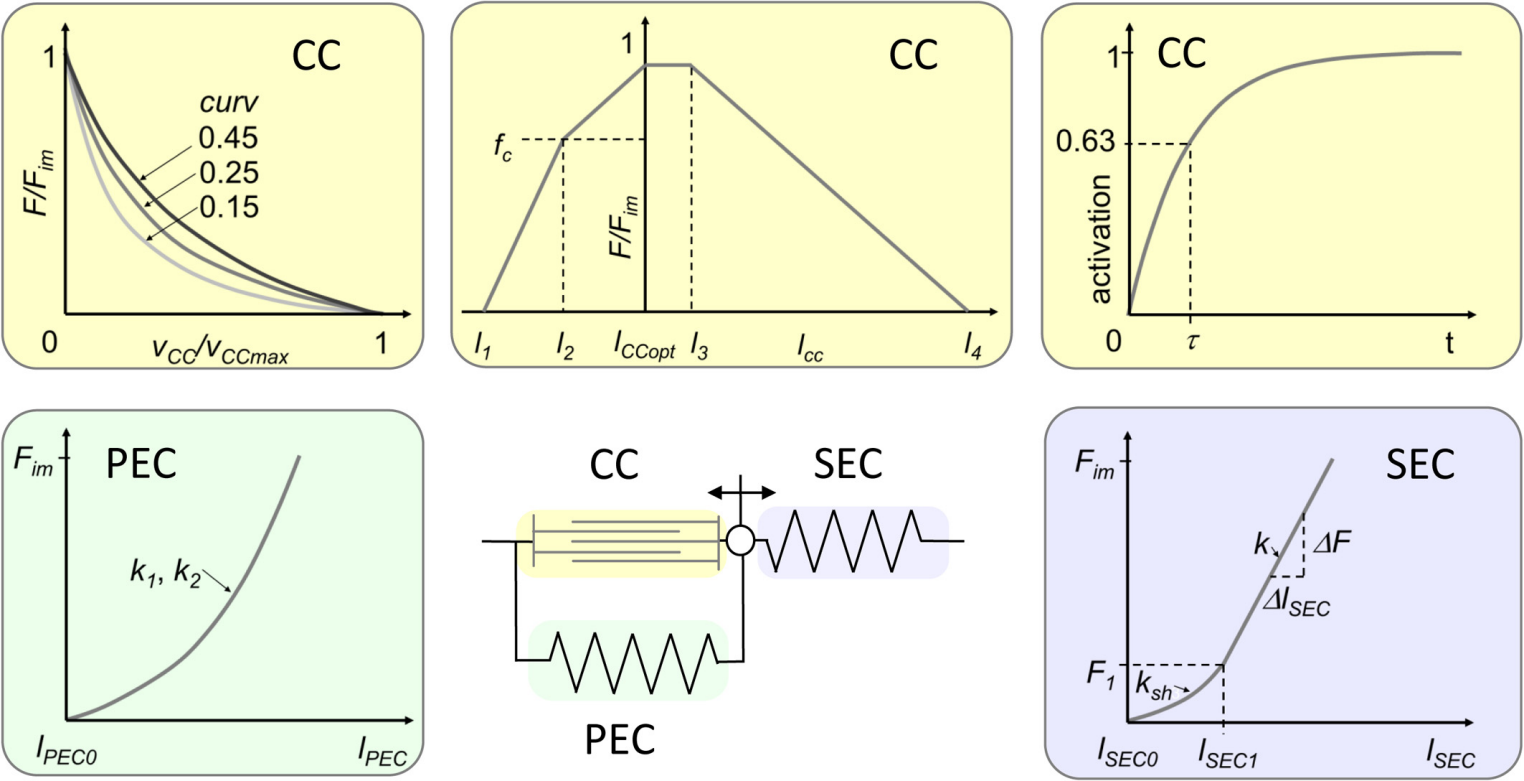
Hill-type muscle model and associated muscle properties. The muscle model [28, 29] for which the parameters are determined in this study consists of a contractile component (CC), a serial elastic component (SEC) and a parallel elastic component (PEC). Muscle components and associated muscle properties (force-velocity relation, force-length relation, activation-time relation, force-elongation relation of SEC and PEC) are marked with the same background color. Corresponding model parameters are explained in section 2.3.

In Hill-type muscle models, the active contractile properties of the fibers are represented by a contractile component (CC). Its length is usually taken to be the mean optimal fiber length, and sometimes the mean pennation angle is considered [6]. Passive tissues in parallel to the fibers like connective tissue and titin (though titin may be considered to be a semi-active element, [27]) can be represented by a parallel elastic component (PEC). Tendon and aponeurosis thought to act in series with the fibers [22] are represented by a serial elastic component (SEC). In the structurally more convincing arrangement of these three components [28] the SEC is in series to both the CC and the PEC (Fig 1). Typical constitutive functions describing the components are depicted in Fig 1.

Since more than 60 years it is known that muscle force further depends on contraction history [30]. For example, force is enhanced in the isometric phase following active stretching (force enhancement, FE) and depressed following active shortening (force depression, FD) compared with the corresponding isometric muscle force. Force enhancement effects can be much larger (2 *F*_*im*_ at lengths with no filament overlap, [31]) than force depression effects (0.05–0.2 *F*_*im*_, [30, 32]). Currently, the causes of these history effects remain a matter of scientific debate [8, 33–35]. Discussed mechanisms are e.g. the contribution of half-sarcomere chain dynamics [34, 36, 37] or non-cross-bridge contributions to muscle tension [27, 31, 38]. Muscle specific differences in contraction history are rarely examined, particularly as experimental protocols and conditions differ, which hampers comparison.

The calf musculature is frequently used as a research object in muscle experiments and simulations (gastrocnemius (GAS), soleus (SOL) and/or plantaris (PLA); e.g. [21, 39–41]). These distal muscles enable comparably easy surgical access, and their long distal tendons simplify fixation to force measurement equipment in animal models. Some of these muscles were observed in recent in vivo studies with respect to their 3D behavior [42, 43]. Understanding e. g. 3D muscle deformations and related force effects in muscle packages by neuromuscular simulations requires consistent specific muscle properties and muscle architectures. Gained insights into fundamental 3D muscle functions may then be generalized which makes the collection of comprehensive data sets important from a clinical perspective. To our knowledge, such comprehensive consistent muscle properties are not available for the calf musculature.

**Fig 2:**
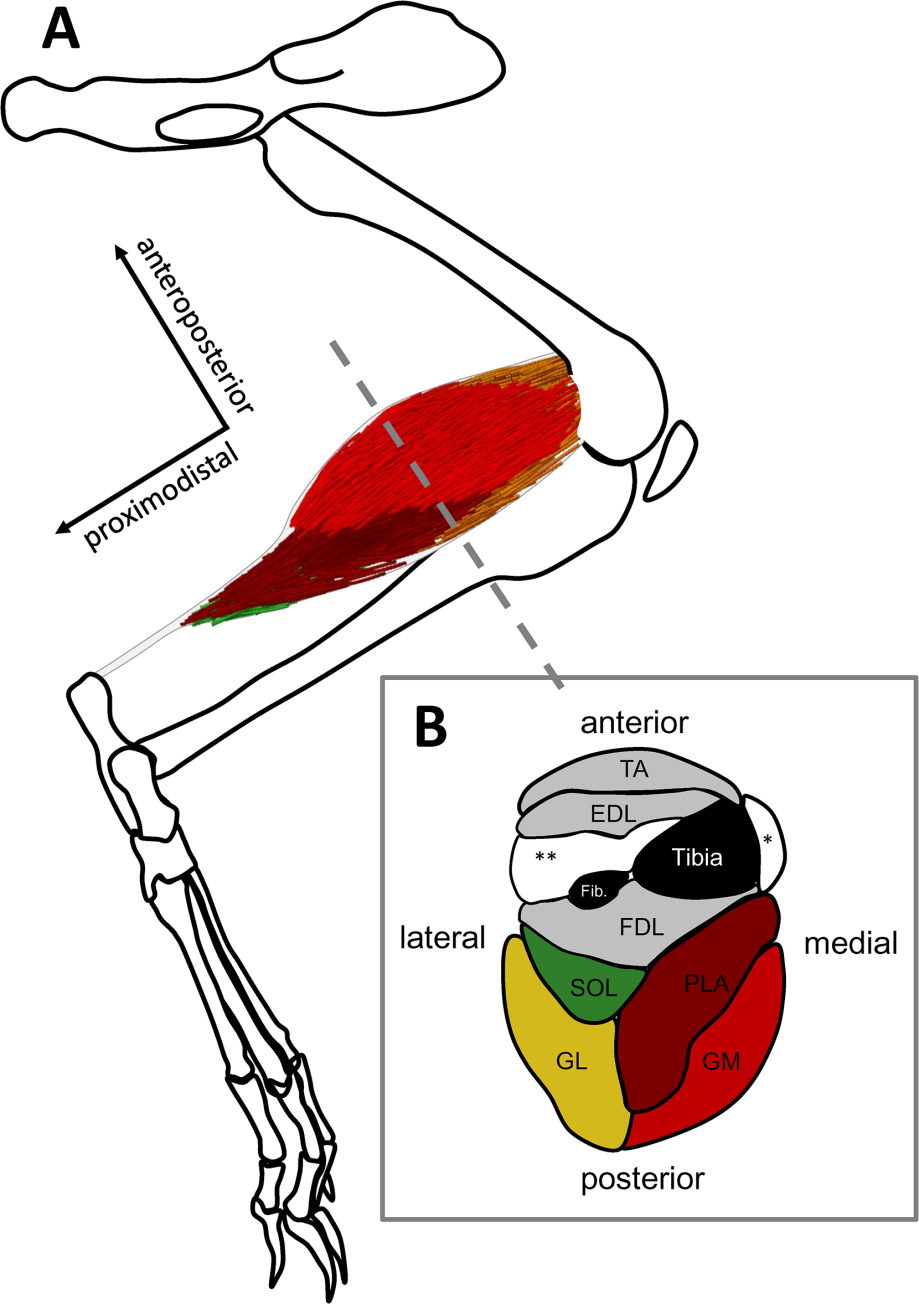
Schematic of the rabbit calf muscles. (A) Medial view of the left pelvic limb and the calf muscles whose dynamic muscle properties and architecture have been determined (GAS, PLA, SOL). The grey dashed line marks the transversal cross-section of the limb shown in (B). For the grey muscles (FDL, EDL, and TA), only dynamic muscle properties were determined (see Supporting Information, S1 Text). White muscles (**peronaei muscles, * *M*. *extensor hallucis longus*) were not examined. The axes are shown for orientation.

The aim of this study is to provide comprehensive data sets of specific muscle properties (force-length relation, force-velocity relation, force-strain relations of SEC and PEC, activation time constant) and the 3D architecture of the superficial rabbit calf muscles for future research. To achieve this, we determine muscle properties of GAS, PLA, and SOL in *in situ* experiments (*n* = 6 per muscle) and measure the 3D architecture by manual digitization ([16]; *n* = 3 per muscle). Because there is no generally accepted model of history effects, we provide standardized data for force enhancement and force depression (*n* = 3 per muscle) which can be used to adapt parameters of custom models describing these effects. Striving for completeness with respect to the shank musculature (Fig 2), we provide the muscle properties for further shank muscles (flexor digitorum longus (FDL), extensor digitorum longus (EDL) and tibialis anterior (TA), *n* = 1).

## Methods

### 2.1 Experimental setup

Experiments on female New Zealand white rabbits (*Oryctolagus cuniculus*, *n* = 21, age: about 16 weeks) with an average weight of 3.37 ± 0.51 kg (mean ± SD, Table 1) were carried out in strict accordance with the recommendations of the German animal welfare law (Tierschutzgesetz, BGBl. I 1972, 1277, section 8). The protocol of this study was approved by the competent authority for animal welfare in Thuringia, Germany (Landesamt für Verbraucherschutz (Abteilung Gesundheitlicher und technischer Verbraucherschutz); Permit Number: 02-022/11 and 02-027/14). All experiments were performed under anesthesia with natrium pentobarbital (Nembutal, 80 mg/kg body weight) and Bupivacain (1 ml, 0.5%, epidural), and all efforts were made to minimize suffering. Experimental setup, anesthesia and preparation of rabbit SOL have been described earlier [43]. Procedures were similar for all muscles analyzed in this study (Table 1). In short, the specific muscle was freed from its surrounding tissues and the rabbit was fixed by clamping hip, knee and ankle with three pairs of bone pins. The distal tendon was attached horizontally to a muscle lever system (Aurora scientific 310B-LR). The sciatic nerve was stimulated (Aurora Scientific 701C) with 100 μs square wave pulses at 100–140 Hz (supramaximal tetanic muscle stimulation). Body temperature was maintained at 39°C using a heating pad. The muscle was sprinkled with heated (39°C) physiological saline solution during the entire experiment.

**Table 1:** Specifications of observed muscles.

Muscle	GAS	PLA	SOL	FDL	EDL	TA
Dynamic muscle properties (*n*)	6	6	6	1	1	1
animal mass [kg]	3.02 ± 0.39	3.27 ± 0.22	3.89 ± 0.47	2.76	3.52	3.38
muscle mass [g]	16.04 ± 1.32	6.31 ± 0.74	3.26 ± 0.32	4.78	4.77	3.52
*L*_*MTC_0*_ [mm]	118.2 ± 6.2	112.0 ± 6.6	102.9 ± 2.0	104.2	103.4	91.9
3D muscle architecture (*n*)	3	3	3	N/A	N/A	N/A
animal mass [kg]	3.28 ± 0.17*	3.28 ± 0.17*	3.28 ± 0.17*	N/A	N/A	N/A

Muscle and animal mass as well as the muscle-tendon complex length *L*_*MTC_0*_ measured at ankle and knee joint angles of 90° (cf. Fig 1). *n*: number of muscles.

*Architecture of GAS, PLA, and SOL was determined from the left legs of three rabbits.

### 2.2 Experiments for determination of muscle properties

First, the muscle-tendon complex length (*L*_*MTC_0*_) was measured *in situ* with a micrometer at an ankle and knee joint angle of 90° (Table 1). To determine the specific muscle properties (force-length relation, force-velocity relation, force-strain relation of the SEC and PEC, and activation time constant), isometric, isotonic and isokinetic contractions were performed. We identified the force-velocity relation by a series of about 10 isotonic contractions against forces in the range of 0.1 *F*_*im*_ to 0.9 *F*_*im*_. Similar to [44], the force-strain relation of the SEC was obtained from the length tension data of a quick (exceeding maximum contraction velocity *v*_*CCmax*_) isokinetic contraction accounting for fiber shortening. The active force-length relation of the CC and the force-strain relation of the PEC were determined from a series of 15–20 isometric contractions (with length increments of 1–2 mm) considering the interaction of the passive elastic structures [28]. The activation parameter *τ*, describing calcium concentration and thus muscle activation, was determined from an isometric contraction at optimal muscle length using a Hill-type model [29]. For details of these parameter determinations, see [29, 45].

History effects were identified for GAS, PLA, and SOL (n = 3 per muscle) using isokinetic ramps [32] with three different shortening / lengthening velocities (0.35, 0.7, and 1.4 *l*_*fm*_/s, where *l*_*fm*_ is the mean fascicle length determined by manual digitization; see section 2.4), and a ramp length of 0.3 *l*_*fm*_. The isokinetic ramp started after an isometric pre-contraction (200 to 400 ms) which was sufficient to reach maximum isometric force. After the end of the ramp, stimulation continued (SOL: 1300 ms; other muscles: 500 ms) to allow sufficient force recovery during the subsequent isometric phase. Longer continued stimulation was used for SOL because this slow twitch fibered muscle [46] is more fatigue resistant than the other muscles. Shortening and lengthening ramps for determination of force depression and force enhancement started at optimum muscle length plus 0.15 *l*_*fm*_ and minus 0.15 *l*_*fm*_, respectively. Force depression and force enhancement were identified as the difference in force between the force at the end of the ramp experiment and the force at the end of an isometric reference contraction of same duration at the same target length. For FDL, EDL, and TA no muscle architecture and thus no mean fascicle length (*l*_*fm*_) was determined. Thus, for these muscles isokinetic ramps had velocities of 5, 10 and 20 mm/s starting at optimum muscle length plus 2 mm (force depression) and minus 2 mm (force enhancement) with a ramp length of 4 mm.

### 2.3 Hill-type muscle model

Parameters were determined for a typical Hill-type muscle model [29] consisting of a PEC in parallel to the CC, and a SEC connected to both of them. Using this model, the active force generated by the contractile component *F*_*cc*_ is the difference between the single forces in the SEC and the PEC and can be described by a typical product approach [6]:

$$F_{CC}=F_{SEC}-F_{PEC}=A\cdot F_{im}\cdot f_{l}(l_{CC})\cdot f_{v}(v_{CC}).$$ (1)

In Eq (1), *A* is the muscle activation and *f*_*l*_ as well as *f*_*v*_ are factors that describe the force–length and force–velocity relations normalized to *F*_*im*_, respectively.

The CC force-length relationship was described with a piecewise linear equation

$$f_{l}(l_{CC})=\{\begin{array}{l} \;\frac{f_{c}}{l_{2}-l_{1}}\cdot (l_{CC}-l_{1}),\;l_{1}\leq l_{CC}\leq l_{2}\\ \;f_{c}+\frac{f_{c}-1}{l_{2}}\cdot (l_{CC}-l_{2}),\;l_{2}<l_{CC}\leq l_{CCopt}\\ \;1,\;l_{CCopt}<l_{CC}\leq l_{3}\\ \;1+\frac{-1}{l_{4}-l_{3}}\cdot (l_{CC}-l_{3}),\;l_{3}<l_{CC}\leq l_{4} \end{array}$$ (2)

where *f*_*c*_ is the force at which the ascending limb changes slope, *l*_*CC*_ is the CC length, *l*_*CCopt*_ the optimal CC length and *l*_*1*_, *l*_*2*_, *l*_*3*_, *l*_*4*_ are specific lengths that are crucial for the sarcomere force-length relationship (Fig 1).

The force-velocity relationship

$$f_{v}(v_{CC})=\frac{v_{CCmax}-v_{CC}}{v_{CCmax}+v_{CC}/curv}\;v_{CC}<0,$$ (3)

follows the Hill hyperbola [5] for concentric contractions, with *v*_*CCmax*_ being the maximal CC shortening velocity, and *curv = a/F*_*im*_ (damping increases with decreasing *curv*, see Fig 1; *a* describes the force asymptote [5]) is an inverse measure of the relation’s curvature.

The latency between the supramaximal stimulation and the muscle activation was modelled as a first order linear differential equation [47]

$$\frac{dA}{dt}=\frac{1-A}{\tau }$$ (4)

where the activation parameter *τ* lumps the time constants of calcium influx from the sarcoplasmic reticulum into the sarcoplasm, and *A*(*t* = 0) = 0.

The SEC force-elongation relationship *F*_*SEC*_(Δ*l*_*SEC*_) was taken from [6]

$$\begin{array}{l} F_{SEC}(\Delta l_{SEC})=\frac{F_{1}}{e^{k_{sh}}-1}\cdot \left(e^{\frac{k_{sh}\cdot \Delta l_{SEC}}{\Delta l_{SEC1}}}-1\right),\;0<\Delta l_{SEC}<\Delta l_{SEC1}\\ F_{SEC}(\Delta l_{SEC})=F_{1}+k\cdot (\Delta l_{SEC}-\Delta l_{SEC1}),\;\Delta l_{SEC1}\leq \Delta l_{SEC} \end{array}$$ (5)

where *Δl*_*SEC1*_ and *F*_*1*_ are the elongation and the force at which the force-elongation relation changes from exponential to linear. *k* was calculated from *Δl*_*SEC1*_, *F*_*1*_, the dimensionless shape parameter *k*_*sh*_ and the constraint that stiffness at Δ*l*_*SEC*_ = Δ*l*_*SEC*1_ is the same for each equation.

A PEC force-elongation relation

$$F_{PEC}(\Delta l_{PEC})=k_{1}\cdot \left(e^{k_{2}\cdot \Delta l_{PEC}}-1\right),\;if\;\Delta l_{PEC}>0$$ (6)

depending on *k*_*1*_ and *k*_*2*_ was taken from [48].

### 2.4 Determination of muscle architecture

Muscle architecture of GAS, PLA, and SOL was determined from the left legs of three rabbits (R1 to R3, *m* = 3.28 ± 0.17 kg) by manual digitization [16, 43]. After the rabbit was killed with an overdose of pentobarbital, the leg was amputated above the knee. The skin was removed and the preparation fixed in Bouin solution (an aqueous solution of picric acid, acetic acid and formaldehyde minimizing tissue shrinkage [49, 50]) for 48 h [14] at knee and ankle angles of about 79° and 93°, respectively. Subsequently, the bone–muscle preparation was cast in wax to provide additional mechanical stability during the digitizing process. For the digitization of the whole muscle architecture of each muscle, small fascicle bundles were successively dissected with a micro forceps. Their original position was then recorded using a manual 3D digitizer (MicroScribe MLX) with a sampling frequency of 5 Hz and an accuracy of 0.07 mm. This process was repeated until all fascicles of the individual muscle were recorded. Each fascicle was described by 20 points. During the dissection and the digitizing, the palm of the hand holding the digitizer-handpiece was placed on the preparation-table to minimize movement of the digitizer tip (< 0.1 mm). Fascicle length and pennation angle were calculated as described in [16]. In addition to recording the fascicle bundle positions, the insertions and origins of the muscles were recorded for each animal (S1, S5, and S9 Datasets).

### 2.5 Statistics

Prior to analysis, muscle parameters were normalized using pertinent units. Parameters were tested for normal distribution using the Kolmogorov-Smirnov-Test with Lilliefors correction. All data were normally distributed. The Levené test was used to check variance homogeneity. To test whether muscle properties differ between the muscles (GAS, PLA, SOL) an analysis of variance (ANOVA) was calculated. In case that the ANOVA demonstrated significant main effects, post hoc analyses were performed using the Tukey HSD test if variances were homogenous. Otherwise the Tamhane test was used. The significance level was set at *P* < 0.05. All analyses were performed using SPSS 22 (IBM Corp, Armonk, NY, USA). The effect sizes *f* were calculated as

$$f=\frac{\sigma_{m}}{\sigma },$$ (7)

where σ_m_ is the standard deviation of the population means, and σ the within-population standard deviation [51]. The effect sizes were classified as low (*f* = 0.1), medium (*f* = 0.25) and large (*f* > 0.40) [51].

## Results

### 3.1 Muscle properties

**Fig 3:**
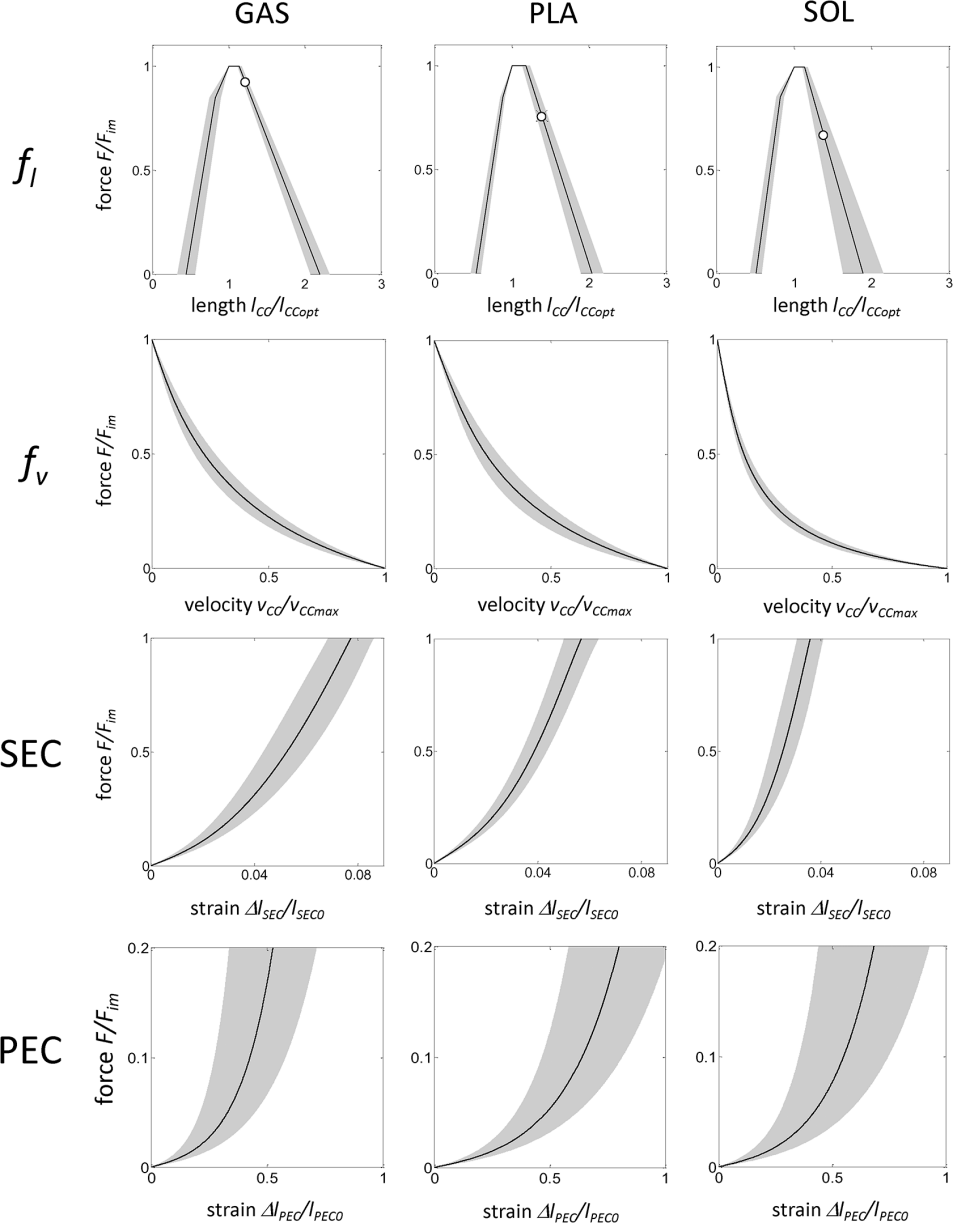
Muscle properties of GAS, PLA, and SOL. The black curves indicate mean values, whereas the grey areas depict the standard deviations. First row: force–length (*f*_*l*_) relation. *F*_*im*_ is the maximum isometric muscle force, *l*_*CC*_ and *l*_*CCopt*_ are the length and the optimal length of the contractile component, respectively. To avoid muscle damage, the muscles were lengthened until passive forces reached about 0.2 *F*_*im*_ (marked with a white circle). Second row: force–velocity (*f*_*v*_) relation. *v*_*CCmax*_ is the maximal shortening velocity of the contractile component. Third row: Force–strain relation of the series elastic component (SEC). *Δl*_*SEC*_ and *l*_*SEC0*_ are the length change and the slack length of the series elastic component, respectively. Last row: Force–strain relation of the parallel elastic component (PEC). *Δl*_*PEC*_ and *l*_*PEC0*_ are the length change and the slack length of the parallel elastic component, respectively.

Active and passive muscle properties vary considerably between GAS, PLA, and SOL. We found significant differences for the parameters *l*_*CCopt*_, *v*_*CCmax*_, *curv*, *Δl*_*SEC1*_/*l*_*SEC0*_, *k*, *l*_*SEC0*_, and *l*_*PEC0*_ (Table 2). All experimental force–velocity relationships feature the typical hyperbolic shape (Fig 3, second row) observed by [5]. Maximum shortening velocity of GAS (13.5 ± 1.7 *l*_*CCopt*_/s) is about twice the value of SOL (6.4 ± 1.0 *l*_*CCopt*_/s). Maximum shortening velocity of PLA (10.1 ± 3.3 *l*_*CCopt*_/s) is in between these values. The *curv* values of the force-velocity relation are similar for GAS and PLA (0.47 ± 0.09 and 0.41 ± 0.16, respectively) but about three times the value for SOL (0.15 ± 0.05). The calf muscles exhibited a characteristic force–length dependency (Fig 3, upper row) which is attributable to the muscle fiber force–length relationship [12]. Maximum isometric forces produced at optimum muscle lengths by GAS, PLA, and SOL are 161.3 ± 18.2 N, 86.4 ± 21.3 N, and 24.1 ± 5.8 N, respectively. Considering a muscle tissue density of 1.056 g/cm^3^ [52], as well as a mean muscle mass (Table 1) and mean optimal fiber length (Table 2), the cross-sectional area (CSA) can be calculated (GAS: 8.63 cm^2^, PLA: 4.57 cm^2^, SOL: 1.44 cm^2^). This leads to similar mean muscle stresses of 18.9, 18.8, and 17.0 N/cm^2^ for GAS, PLA, and SOL, respectively. Optimum fiber lengths of SOL (22.1 ± 4.5 mm) are longer than those of PLA (13.2 ± 1.3 mm) enabling a much larger working range of SOL. Series and parallel elastic components possess typical [6] nonlinear force–strain characteristics (Fig 3). The standard deviations of the determined muscle properties are small, with the exception of the force–strain relation of the parallel elastic component (Fig 3, bottom row) which is about three times the standard deviation observed for the SEC.

**Table 2:** Muscle parameters of GAS, PLA, and SOL.

	Muscle	GAS	PLA	SOL		
	Parameter	mean ± S.D.	mean ± S.D.	mean ± S.D.	p	f-value
*f*_*l*_	*l*_*1*_ */l*_*CCopt*_	0.44 ± 0.11	0.53 ± 0.06	0.50 ± 0.05	ns	-
	*l*_*2*_ */l*_*CCopt*_	0.82 ± 0.07	0.88 ± 0.04	0.82 ± 0.03	ns	-
	*l*_*3*_ */l*_*CCopt*_	1.14 ± 0.03	1.19 ± 0.07	1.14 ± 0.04	ns	-
	*l*_*4*_ */l*_*CCopt*_	2.21 ± 0.21	2.05 ± 0.21	1.95 ± 0.42	ns	-
	*f*_*c*_ [*F*_*im*_]	0.85 ± 0.08	0.85 ± 0.07	0.85 ± 0.05	ns	-
	*F*_*im*_ / CSA [N/cm^2^]	18.9 ± 3.3	18.8 ± 3.1	17.0 ± 4.3	ns	-
	*l*_*CCopt*_ [mm]	17.7 ± 1.1	13.2 ± 1.3	22.1 ± 4.5	0.01 (*,†)	1.30
*f*_*v*_	*v*_*CCmax*_ [*l*_*CCopt*_/s]	13.5 ± 1.7	10.1 ± 3.3	6.4 ± 1	0.01 (†,#)	1.29
	*curv*	0.47 ± 0.09	0.41 ± 0.16	0.15 ± 0.05	0.01 (†,#)	1.25
*A*	*τ* [s]	0.06 ± 0.03	0.06 ± 0.03	0.04 ± 0.01	ns	-
*SEC*	*F*_*1*_ /*F*_*im*_	0.31 ± 0.06	0.31 ± 0.09	0.43 ± 0.14	ns	-
	*Δl*_*SEC1*_/*l*_*SEC0*_	0.049 ± 0.009	0.036 ± 0.006	0.026 ± 0.004	0.00 (*,#)	1.42
	*k*_*sh*_	2.2 ± 0.3	2.6 ± 0.5	2.7 ± 0.7	ns	-
	*k* [N/mm]	30.3 ± 1.4	21.9 ± 4.9	14.1 ± 2.7	0.00 (*,†,#)	2.01
	*l*_*SEC0*_ [mm]	105.3 ± 5.2	102 ±5.6	86.9 ± 2.7	0.00 (†,#)	1.79
*PEC*	*k*_*1*_ [N]	0.048 ± 0.057	0.114 ± 0.171	0.034 ± 0.031	ns	-
	*k*_*2*_ [mm^-1^]	0.50 ± 0.18	0.47 ± 0.17	0.35 ± 0.12	ns	-
	*l*_*PEC0*_ [mm]	12.9 ± 2.9	9.8 ± 1.9	16.0 ± 1.8	0.00 (*,†,#)	1.41
*FD*	FD_0.35_ [%*F*_*im*_]	17.3 ± 0.9	17.8 ± 4.8	9.7 ± 1.2		
	FD_0.7_ [%*F*_*im*_]	16.6 ± 0.7	15.4 ± 4.0	8.0 ± 0,9		
	FD_1.4_ [%*F*_*im*_]	16.0 ± 0.7	14.6 ± 3.9	5.8 ± 1.5		
*FE*	FE_0.35_ [%*F*_*im*_]	7.7 ± 1.6	17.1 ± 9.6	11.3 ± 1.5		
	FE_0.7_ [%*F*_*im*_]	7.7 ± 1.4	17.0 ± 9.8	11.3 ± 1.7		
	FE_1.4_ [%*F*_*im*_]	7.7 ± 1.6	17.0 ± 8.7	11.2 ± 1.3		

Mean and standard deviation of muscle specific properties; *f*_*l*_: force-length relation, *f*_*v*_: force-velocity relation, *A*: muscle activation, SEC: series elastic component, PEC: parallel elastic component. Force depression (FD) and force enhancement (FE) were determined for three different velocities (0.35, 0.7, and 1.4 mean fascicle lengths per second). Significant differences are marked as follows

* p < 0.05 between GAS and PLA

† p < 0.05 between PLA and SOL

# p < 0.05 between SOL and GAS.

ns means not significant. The effect sizes were classified as low (*f* = 0.1), medium (*f* = 0.25) and large (*f* > 0.40) [51]. No statistics was performed for FD and FE due to small sample size (*n* = 3).

**Fig 4:**
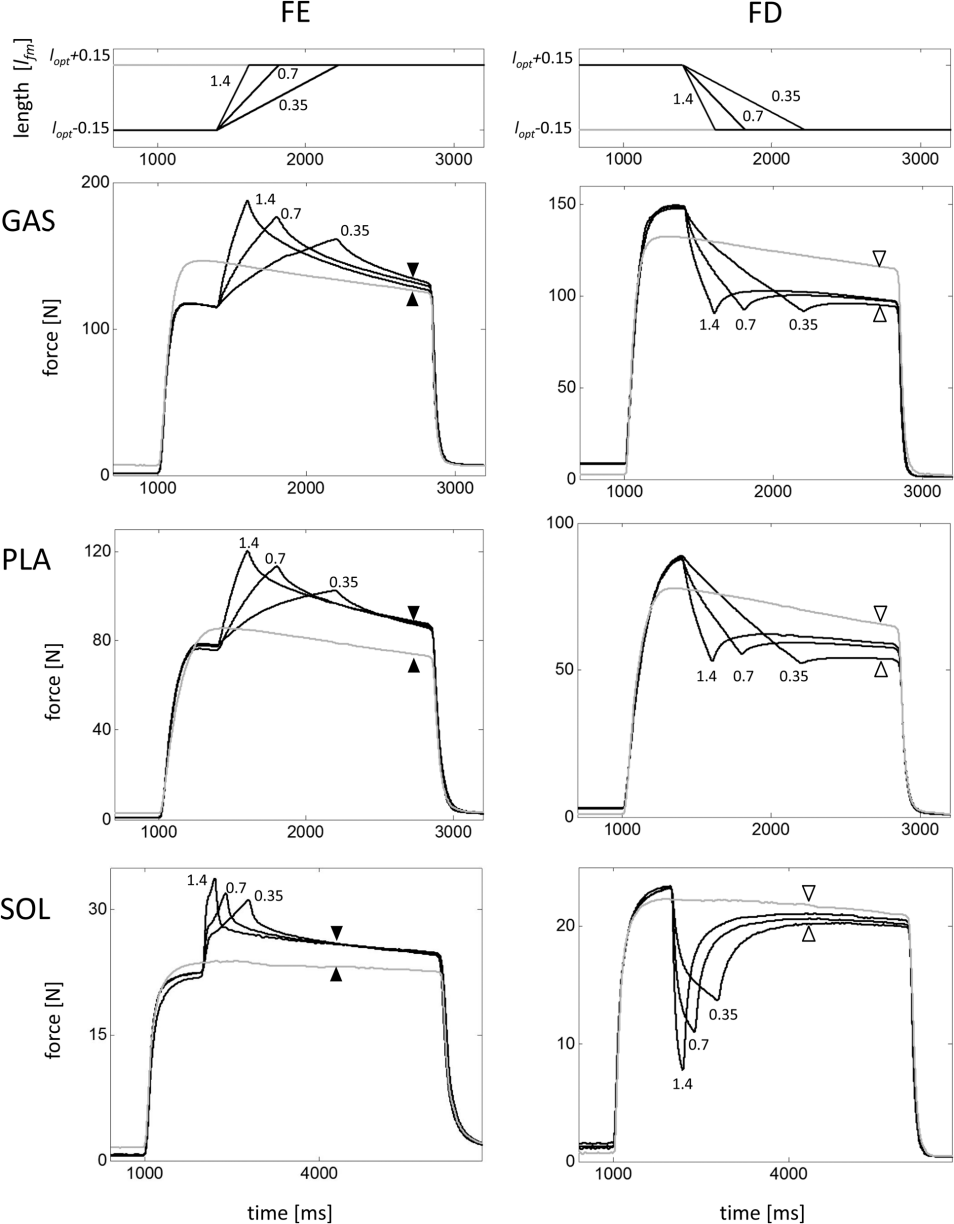
Force enhancement (FE) and force depression (FD) experiments. Typical experiments are shown for one GAS (*m* = 14.8 g), SOL (*m* = 3.3 g), and PLA (*m* = 7.5 g), respectively. Exemplary isokinetic ramps are depicted for GAS in the top row; numbers without units indicate velocity in mean fascicle lengths per second. FE (difference between black triangles) and FD (difference between white triangles) are the force difference between ramp experiment (black) and isometric reference contraction (grey) determined 500ms (GAS, PLA) and 1300ms (SOL) after the end of the ramp, shown exemplarily for the slowest (0.35 *l*_*fm*_/s) ramp.

Forces of GAS, PLA, and SOL were enhanced following stretching and were depressed following shortening compared with the corresponding isometric forces (Table 2, Fig 4). For all muscles, force depression was inversely related to the ramp velocity (Table 2). This effect was pronounced for the SOL. In contrast, force enhancement was independent of ramp velocity. The magnitude of force enhancement increased from GAS (≈ 8% *F*_*im*_), to SOL (≈ 11% *F*_*im*_) up to PLA (≈ 17% *F*_*im*_). At the slowest ramp velocity (0.35 *l*_*fm*_/s), force depression of GAS and PLA (≈ 17.5% *F*_*im*_) was about two fold that of SOL (9.7% *F*_*im*_).

We report additionally obtained muscle properties of further shank muscles (FDL, EDL, TA; *n* = 1) for completeness in the Supporting Information (S1 Text).

### 3.2 Architecture

**Fig 5:**
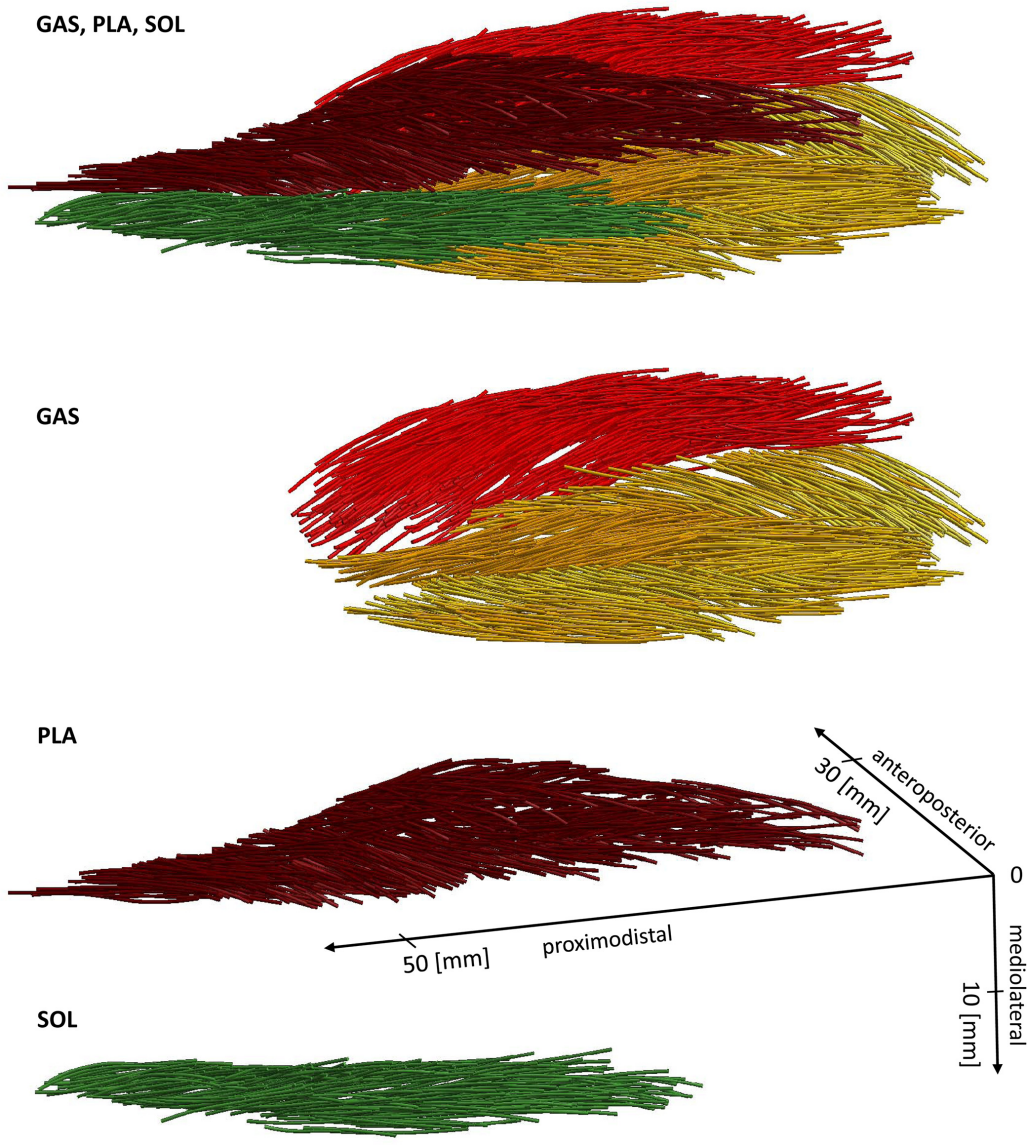
Muscle architectures of GAS, PLA, and SOL of R1 left pelvic limb. Muscle fascicles of GAS medialis and lateralis are shown in light red and yellow, respectively. The proximodistal axis corresponds to the mean force axis of the calf muscles, running from mean muscle origin at the humerus to the insertion at the calcaneus. The corresponding 3D data of the muscle fascicles are provided in the Supporting Information (S2–S4 Datasets).

General architectural properties of GAS, PLA, and SOL are listed in Table 3. Spatial coordinates of the fascicles of rabbit R1 are presented in Fig 5. Three dimensional fascicle data including origin and insertion of the GAS, PLA, and SOL of the three animals (R1, R2, R3) are provided in txt-format in the Supporting Information (S1–S12 Datasets). SOL (Fig 5, green fascicles) exhibits simple unipennate muscle architecture while GAS (*medialis*: light red fascicles; *lateralis*: yellow fascicles) and PLA (dark red fascicles) show more complex bipennate muscle architectures. For each animal, the mean pennation angles of all three muscles (GAS, PLA, and SOL) were almost similar (Table 3). Differences appear to be about 1–2°, only. In between the different animals, variations were slightly larger. Mean pennation angles in R1 are about 5° larger than in R3. These variations in pennation angles have only small (< 0.02 *F*_*im*_) impact on the calculation of muscle force. Mean fascicle lengths (*l*_*fm*_) are larger for the heavier animals (R2 and R3). For each specific animal, GAS and SOL exhibit about the same mean fascicle lengths but in general PLA is about 30% shorter.

**Table 3:** Muscle architecture of GAS, PLA, and SOL.

animal	muscle	mean pennation angle [°]	mean fascicle length *l*_*fm*_ [mm]	ankle joint angle [°]	knee joint angle [°]
R1 (m = 3.04 kg)	GAS	16.6 ± 7.3	14.1 ± 2.2	94	75
	PLA	16.1 ± 6.2	10.8 ± 2.1	94	75
	SOL	15.8 ± 5.4	14.0 ± 2.2	94	75
R2 (m = 3.10 kg)	GAS	14.5 ± 6.4	17.2 ± 3.3	93	91
	PLA	14.8 ± 6.3	11.1 ± 2.5	93	91
	SOL	12.0 ± 3.6	17.0 ± 2.2	93	91
R3 (m = 3.10 kg)	GAS	12.3 ± 5.2	19.3 ± 3.2	92	70
	PLA	11.1 ± 4.8	14.4 ± 3.1	92	70
	SOL	11.3 ± 3.5	20.4 ± 3.1	92	70

Mean pennation angle and fascicle length determined from the left legs of three rabbits (R1, R2, R3) by manual digitization. 1838, 1773, and 1523 fascicles have been digitized for R1, R2, and R3, respectively. Their lengths and pennation angles are normally distributed. The muscle architecture is exemplarily shown for R1 in Fig 5. Note that the complete 3D data are provided in the Supporting Information (S1–S12 Datasets).

## Discussion

Experiments performed within this study provide comprehensive data sets for the rabbit calf muscles GAS, PLA, and SOL consisting of manually digitized 3D muscle architectures and specific muscle properties including the quantification of history effects.

### 4.1 Comparison with literature: muscle properties

Mean muscle tensions of GAS, PLA, and SOL determined in this study are comparable to values between 14 and 20 N/cm^2^ observed for other small mammal muscles, e.g. rat SOL [53, 54], rabbit SOL [43], rat GAS medialis [45], kangaroo rat PLA [55], or guinea pig SOL [53].

SOL maximum shortening velocity and *curv* value of the force–velocity relation (Table 2) are similar to values reported for slow twitch muscles (*v*_*CCmax*_: 3–7 *l*_*CCopt*_/s, *curv*: 0.1–0.2, [13, 29, 56]). Higher shortening velocities and *curv* values of GAS and PLA agree with values reported for fast twitch muscles (*v*_*CCmax*_: 9–20 *l*_*CCopt*_/s, *curv*: 0.3–0.5, [13, 56, 57]). These results are in agreement with fiber type compositions of rabbit GAS (> 75% fast twitch fibers), PLA (> 90% fast twitch fibers), and SOL (> 99% slow twitch fibers; [46, 58]).

The active force-length relation was described by the theoretical sarcomere force–length relationship [12]. As demonstrated in recent studies [28, 59] this relation enabled the accurate prediction of experimental rabbit and cat muscle forces. Winters [59] reproduced the active force-length relations of rabbit TA, EDL, and extensor digitorum II based on myofilament lengths using a scaled sarcomere model. In contrast, we fitted the experimental force-length data using a piecewise linear equation. However, the results are consistent for the most part. Starting from optimum muscle length, the rabbit muscles were able to shorten by 50% in both studies. For lengthening muscle, force production is limited to 1.6 *l*_*CCopt*_ in the scaled sarcomere model, but reaches about 2 *l*_*CCopt*_ for GAS, PLA, and SOL (Fig 3). Differences might be due to more complex muscle architectures, especially of GAS and PLA (Fig 5, see Sect. 4.2), influencing the width of the force-length relation [60]. Also, to avoid muscle damage induced by high passive forces, muscles in our study were lengthened only up to passive forces of about 0.2 *F*_*im*_ (Fig 3, upper row, marked by a white circle). Thus, the slope of the descending limb of the force length relation was determined using limited experimental data and should be considered with caution. The change in slope at the ascending limb of the force-length relation is fixed at 0.7 *F*_*im*_ in the scaled sarcomere model, but appears at higher forces (*f*_*c*_ = 0.85 *F*_*im*_) in our measurements. However, the change in slope of experimental TA force-length relations appears at higher forces in the study of [59] (their Fig 3A) which is in agreement with our observations on TA (S1 Table).

Series elastic structures exhibit a typical [6] nonlinear force-strain relationship (Fig 3, third row). The mean maximum strain at *F*_*im*_ was 5.5% and 3.6% *L*_*SEC0*_ for PLA and SOL (Fig 3), respectively, which is expected for tendinous tissue [61, 62]. The SEC of the GAS was more compliant (7.7% strain at *F*_*im*_). This may be due to a higher proportion of aponeuroses in the muscle tendon complex which may be more compliant than tendons [63].

The standard deviations of the determined muscle properties are small, with the exception of the force–strain relation of the parallel elastic component (Fig 3, lower row). This has also been reported for other muscles [43, 64, 65] and may be related to variations in connective tissues (fascia, epimysium, perimysium, endomysium) or titin-isoforms (e.g. [66]).

The behavior of the rabbit muscles observed in this study is mostly consistent with history effects observed in other muscles. As found in our study, force enhancement is independent of stretch velocity [67], and force depression decreases with increasing ramp velocity [30]. The influence of these history effects on the determination of the muscle properties is discussed in the Supporting Information (S2 Text).

Using the same experimental setup and conditions we found muscle specific differences in the amount and in the ratio of force enhancement and force depression (Table 3, S1 Table). These muscle specific properties might be explained by synthesis of different titin isoforms in different rabbit muscles as reported by [66]. Interestingly, these differences in titin isoforms are not related to the fiber type. Interaction of titin with the actin myofilament is assumed to be responsible for the history dependence of muscle contractions [8, 27]. However, experimental and modeling evidence is necessary to demonstrate the conceivable relation between muscle specific titin isoforms and muscle specific history dependence of muscle contraction.

The mechanism and function of contraction history effects are a matter of scientific debate [8, 33–35]. Force enhancement enables the muscle to withstand high forces during eccentric contractions. Rode [27] suggested that force depression is an unwanted by-product of desired force enhancement, and does not occur in stretch-shortening cycles associated with bouncing gaits. The biarticular PLA has comparably short muscle fibers (Table 2) and long tendons, and is appropriate to work as a spring during hopping [58, 68]. Indeed, PLA exhibits comparably high force enhancement (Table 2) which enables generating high forces during eccentric contractions. However, the primary function of muscles working as motors during locomotion, e.g. the monoarticular SOL, is to produce positive work [41, 69, 70]. For these muscles force depression seems to be counterproductive because it reduces positive work. Indeed, SOL exhibited much lower force depression than GAS and PLA (Table 2). These findings support the idea that history effects represent an adaptation to the specific muscle function [27].

### 4.2 Muscle architecture

Studies providing 3D architectural data of muscle packages consisting of several synergistic muscles are rare. Using diffusion tensor imaging 3D muscle architecture of e.g. the human calf [71], human thigh [72], human forearm [73] and mouse hindlimb muscles [74] have been examined. The architecture of the human back [75] and cavy forelimb muscles [14] was determined by manual digitization. However, to the author’s best knowledge, there is no consistent 3D data set of the complete rabbit calf muscle architecture.

Measurements comparable to our experiments were performed on rabbit SOL [43]. The authors examined ten SOL muscles using manual digitization and reported a mean fascicle length of 16.6 ± 2.6 mm which is similar to our value obtained from three muscles (17.1 ± 2.6 mm). A slightly lower mean pennation angle (9.9 ± 2.8° vs. 13.0 ± 2.0°) might be due to smaller ankle joint angles reported in their study. In agreement with our results, Hiepe [76] reported mean fascicle lengths of 16.2 ± 9.1 mm for rabbit GAS *medialis* using DTI. The higher standard deviation in their study might be due to limitations of the diffusion tensor imaging method, e.g. generated fiber tracts may cross muscle borders due to equally oriented adjacent structures, resulting in artificial fascicle traces that are too long [16, 74].

There are few studies dealing with architectural measurements of the PLA [77–80]. None of them utilizes the rabbit as animal model or provides 3D architectural data what makes a direct comparison of fiber lengths and pennation angle impossible. Savelberg [78] mentioned that the non-parallel arrangement of the aponeuroses inside the rat’s PLA features different pennation angles and various fiber lengths. This is in agreement with our observations of the complex bipennate rabbit PLA muscle architecture.

Models of muscle packages are required to understand transverse interaction of muscles with each other [81–83] and with the skeleton, internal muscle forces, and the influence of muscle architecture on contraction dynamics and muscle deformation. In addition to deviating muscle architectures, observed significant differences in normalized dynamical muscle parameters suggest that it is important to use specific muscle parameters in neuromuscular models aiming at understanding fundamental 3D muscle functions. The revelation of such fundamental 3D muscle functions may be relevant from a clinical perspective when assessing the effects of muscle malfunction e.g. on the stabilization of joints during movement. Moreover, 3D muscle models may contribute to a deeper understanding of widespread diseases as chronic low back pain which are accompanied by several changes in the muscle structure as atrophy [84], steatosis [85] or altered fiber type distribution [86].

## Supporting Information

S2–S12 Datasets (except S5 and S9) can be processed using GID software (CIMNE, Barcelona, Spain).

S1 Dataset Rabbit R1: Origins and insertion of GAS, PLA, and SOL in txt-format. (TXT)

S2 Dataset Rabbit R1: GAS 3D muscle architecture in txt-format. (TXT)

S3 Dataset Rabbit R1: PLA 3D muscle architecture in txt-format. (TXT)

S4 Dataset Rabbit R1: SOL 3D muscle architecture in txt-format. (TXT)

S5 Dataset Rabbit R2: Origins and insertion of GAS, PLA, and SOL in txt-format. (TXT)

S6 Dataset Rabbit R2: GAS 3D muscle architecture in txt-format. (TXT)

S7 Dataset Rabbit R2: PLA 3D muscle architecture in txt-format. (TXT)

S8 Dataset Rabbit R2: SOL 3D muscle architecture in txt-format. (TXT)

S9 Dataset Rabbit R3: Origins and insertion of GAS, PLA, and SOL in txt-format. (TXT)

S10 Dataset Rabbit R3: GAS 3D muscle architecture in txt-format. (TXT)

S11 Dataset Rabbit R3: PLA 3D muscle architecture in txt-format. (TXT)

S12 Dataset Rabbit R3: SOL 3D muscle architecture in txt-format. (TXT)

S1 Table Muscle parameters of FDL, EDL and TA. (DOCX)

S1 Text Muscle properties of flexor digitorum longus (FDL), extensor digitorum longus (EDL), and tibialis anterior (TA). (DOCX)

S2 Text Influence of contraction history on the determination of muscle properties. (DOCX)
