# Supplementary material for: Three-Dimensional Muscle Architecture and Comprehensive Dynamic Properties of Rabbit Gastrocnemius, Plantaris and Soleus: Input for Simulation Studies
Source: PLoS One. 2015 Jun 26;10(6):e0130985. doi: 10.1371/journal.pone.0130985 (PMC4482742; doi:10.1371/journal.pone.0130985)
Supplement: S1 Table — (DOCX) [file pone.0130985.s013.docx]

S1 Table. Muscle parameters of FDL, EDL and TA.

Mean and standard deviation of muscle specific properties; *f_l_*: force-length relation, *f_v_*: force-velocity relation, *A*: muscle activation, SEC: series elastic component, PEC: parallel elastic component. Force depression (FD) and force enhancement (FE) were determined for three different velocities (5, 10, and 20 mm/s marked by indices 5, 10 and 20, respectively).

| **Muscle** | | **FDL** | **EDL** | **TA** |
| --- | --- | --- | --- | --- |
| *f_l_* | *l_1_ /l_CCopt_* | 0.40 | 0.39 | 0.40 |
|  | *l_1_ /l_CCopt_* | 0.78 | 0.82 | 0.80 |
|  | *l_1_ /l_CCopt_* | 1.18 | 1.21 | 1.16 |
|  | *l_1_ /l_CCopt_* | 2.45 | 2.03 | 2.45 |
|  | *f_c_* [*F_im_*] | 0.81 | 0.91 | 0.84 |
|  | *F_im_* / CSA [N/cm^2^] | 13.9 | 13.0 | 21.9 |
|  | *l_CCopt_* [mm] | 10.7 | 14.1 | 36.7 |
| *f_v_* | *v_CCmax_* [*l_CCopt_*/s] | 14.7 | 12.3 | 16.4 |
|  | *curv* | 0.32 | 0.37 | 0.33 |
| *A* | *τ* [s] | 0.07 | 0.07 | 0.01 |
| *SEC* | *F_1_* /*F_im_* | 0.36 | 0.41 | 0.20 |
|  | *Δl_SEC1_*/*l_SEC0_* | 0.032 | 0.035 | 0.027 |
|  | *k_sh_* | 4.3 | 2.0 | 2.1 |
|  | *k* [N/mm] | 36.3 | 15.8 | 9.0 |
|  | *l_SEC0_* [mm] | 95.7 | 90.3 | 56.2 |
| *PEC* | *k_1_* [N] | 0.281 | 0.001 | 0.003 |
|  | *k_2_* [mm^-1^] | 0.22 | 0.82 | 0.49 |
|  | *l_PEC0_* [mm] | 8.5 | 13.1 | 35.7 |
| *FD* | FD_5_ [%*F_im_*] | 30.6 | 19.3 | 25.3 |
|  | FD_10_ [%*F_im_*] | 23.1 | 17.7 | 25.0 |
|  | FD_20_ [%*F_im_*] | 20.0 | 11.3 | 24.8 |
| *FE* | FE_5_ [%*F_im_*] | 38.4 | 22.6 | 25.6 |
|  | FE_10_ [%*F_im_*] | 38.3 | 22.5 | 25.5 |
|  | FE_20_ [%*F_im_*] | 38.4 | 22.4 | 25.4 |
