# Supplementary material for: Three-Dimensional Muscle Architecture and Comprehensive Dynamic Properties of Rabbit Gastrocnemius, Plantaris and Soleus: Input for Simulation Studies
Source: PLoS One. 2015 Jun 26;10(6):e0130985. doi: 10.1371/journal.pone.0130985 (PMC4482742; doi:10.1371/journal.pone.0130985)
Supplement: S1 Text — (DOCX) [file pone.0130985.s014.docx]

**S1 Text. Muscle properties of flexor digitorum longus (FDL), extensor digitorum longus (EDL), and tibialis anterior (TA)**

Muscle properties are given for FDL, EDL, and TA (*n*=1 per muscle) in S1 Table. The curvature parameter *curv* is about 0.35 for all three muscles and thus within the range reported for fast twitch (FT) muscles [[1](#_ENREF_1)]. This is in agreement with the high percentage of FT fibers reported for these rabbit muscles in literature (FDL: ≈ 80 % FT: EDL: > 97 % FT, TA: > 90 % FT, [[2](#_ENREF_2),[3](#_ENREF_3)]. Maximum shortening velocities (FDL: 15 *l_CCopt_*/s, EDL: 12 *l_CCopt_*/s, TA: 16 *l_CCopt_*/s) are comparable to those of the fast twitch fibered GAS. Force enhancement after stretch and force depression after shortening was found in FDL, EDL and TA. As observed for GAS, PLA and SOL (Table 2), force depression decreased with increasing ramp velocity, but force enhancement was independent of ramp velocity. We found highest force enhancement (38.4 % *F_im_*) and force depression (30.6 % *F_im_*) for FDL. At the slowest ramp velocity (5 mm/s) EDL and TA exhibit history effects of about 20 % *F_im_*. Optimum fiber length was two to three times longer for TA (*l_CCopt_* = 36.7 mm) than for EDL and FDL. This is in agreement with reported optimum fiber lengths of 38.5 and 15.3 mm for TA and EDL, respectively [[4](#_ENREF_4)]. Thus TA exhibits the longest working range. Normalized muscle forces (FDL: 13.9 N/cm^2^, EDL 13.0 N/cm^2^) were on the lower range of reported values for small mammals [[1](#_ENREF_1)], whereas TA was able to generate 21.9 N/cm^2^.

**References**

_1. Ranatunga KW, Thomas PE (1990) Correlation between shortening velocity, force-velocity relation and histochemical fibre-type composition in rat muscles. J Muscle Res Cell Motil 11: 240-250._

_2. Wang LC, Kernell D (2001) Fibre type regionalisation in lower hindlimb muscles of rabbit, rat and mouse: a comparative study. J Anat 199: 631-643._

_3. Wank V (1996) Modellierung und Simulation von Muskelkontraktionen für die Diagnose von Kraftfähigkeiten. Köln: Sport und Buch Strauss._

_4. Winters TM, Takahashi M, Lieber RL, Ward SR (2011) Whole muscle length-tension relationships are accurately modeled as scaled sarcomeres in rabbit hindlimb muscles. J Biomech 44: 109-115._
