# Supplementary material for: Three-Dimensional Muscle Architecture and Comprehensive Dynamic Properties of Rabbit Gastrocnemius, Plantaris and Soleus: Input for Simulation Studies
Source: PLoS One. 2015 Jun 26;10(6):e0130985. doi: 10.1371/journal.pone.0130985 (PMC4482742; doi:10.1371/journal.pone.0130985)
Supplement: S2 Text — (DOCX) [file pone.0130985.s015.docx]

**S2 Text. Influence of contraction history on the determination of muscle properties**

History effects impact some of the determined muscle properties. Determination of SEC and PEC parameters is independent of contraction history due to the experimental procedure. The force-length relation of muscle is expected to be underestimated due to shortening of the contractile component against the compliant SEC during otherwise isometric contractions. More specifically, force depression will be maximal for contractions with high forces [[1](#_ENREF_1)] and thus notable SEC strain, i.e. in the plateau region of the force-length relation. Large SEC strain at comparably low forces appears during the onset of isometric contraction, due to the more compliant nonlinear toe region of the SEC force-strain relation [[2](#_ENREF_2)]. Because of these low forces force depression might be marginal in this range [[1](#_ENREF_1)]. Assuming a SEC elongation of about 2 to 2.5 % during the isometric force rise from 0.5 *F_im_* to 1 *F_im_* (for GAS and PLA, Fig. 3) results in fiber shortening of about 0.18 *l_fm_*. Considering maximal force depression of about 17 % induced by a ramp length of 0.3 *l_fm_* (Table 2, GAS and PLA) and a linear relation between ramp length and force depression, maximum isometric force in the plateau region of the force-length relation might be underestimated for GAS and PLA by about 10 %. Applying the same calculation for SOL yields a much smaller underestimation (< 2 %) in maximum isometric force due to lower force depression, stiffer SEC and longer muscle fibers of SOL compared to GAS and PLA (Table 2).

The force-velocity relation was determined from a series of isotonic muscle contractions against varying forces from about 0.1 *F_im_* to 0.9*F_im_*. In one trial the CC first shortens against the SEC during the isometric force rise and subsequently shortens by the same amount like the whole muscle in the isotonic phase because SEC length remains constant. We expect larger force depression effects on the force-velocity relation for higher forces, as force depression depends on force. In the following we estimate the impact of force depression on an isotonic contraction of 0.75 *F_im_* and thus on the force-velocity relation at this point. Force depression in the isometric phase would be half of its value estimated for maximal isometric contraction (see above), i.e. 5 % *F_im_* for GAS and PLA and < 1 % *F_im_* for SOL. In the isotonic phase, maximum shortening velocity (about 0.1 *v_CCmax_*) against an isotonic force of 0.75 *F_im_* was reached after shortening about 0.04 *l_fm_* for PLA and GM. Scaling the observed force depression (Table 2) accordingly, force depression increases by further 2 % *F_im_* in the isotonic phase yielding a total of 7 % *F_im_* for GAS and PLA. For SOL, the estimation yields 2.5 % *F_im_* force depression. Considering the curvature of the force-velocity relation, this results in an underestimation of the contraction velocity of 4 % *v_CCmax_* for GAS and PLA and 2 % *v_CCmax_* for SOL. Hence the force-velocity relation is underestimated, especially at high forces.

To compare history-dependent properties of the rabbit muscles we performed ramp experiments whose ramp lengths and ramp velocities were normalized to the mean fascicle lengths of GAS, PLA, and SOL (Table 3). However, optimal muscle lengths were larger for all muscles (Table 2). Considering these values for normalization, ramps performed for SOL were 25 % shorter than GAS and PLA. Assuming a linear dependence of history effects on ramp length [[3](#_ENREF_3)] this results in an underestimation of SOL history effects of about 25 %. However, for force depression, the associated decreased ramp velocity reduces the expected underestimation. Furthermore, history effects were determined after a longer time period (with respect to the end of the ramp) for SOL than for GAS and PLA. This longer time period was necessary for the slow twitch fibered SOL to allow sufficient force recovery during the isometric phase after the ramp (Fig. 4). SOL properties should be handled with care when comparing history effects amongst the observed muscles.

**References**

_1. Leonard TR, Herzog W (2005) Does the speed of shortening affect steady-state force depression in cat soleus muscle? J Biomech 38: 2190-2197._

_2. Winters JM (1990) Hill-based muscle models: A systems engineering perspective. In: Winters JM, Woo SLY, editors. Multiple Muscle Systems. New York: Springer Verlag. pp. 69-93._

_3. Abbott BC, Aubert XM (1952) The force exerted by active striated muscle during and after change of length. J Physiol 117: 77-86._
